# Supplementary material for: Identification of Target Chicken Populations by Machine Learning Models Using the Minimum Number of SNPs
Source: Animals (Basel). 2021 Jan 19;11(1):241. doi: 10.3390/ani11010241 (PMC7835996; doi:10.3390/ani11010241)
Supplement: Supplementary file 1 [file animals-11-00241-s001.pdf]

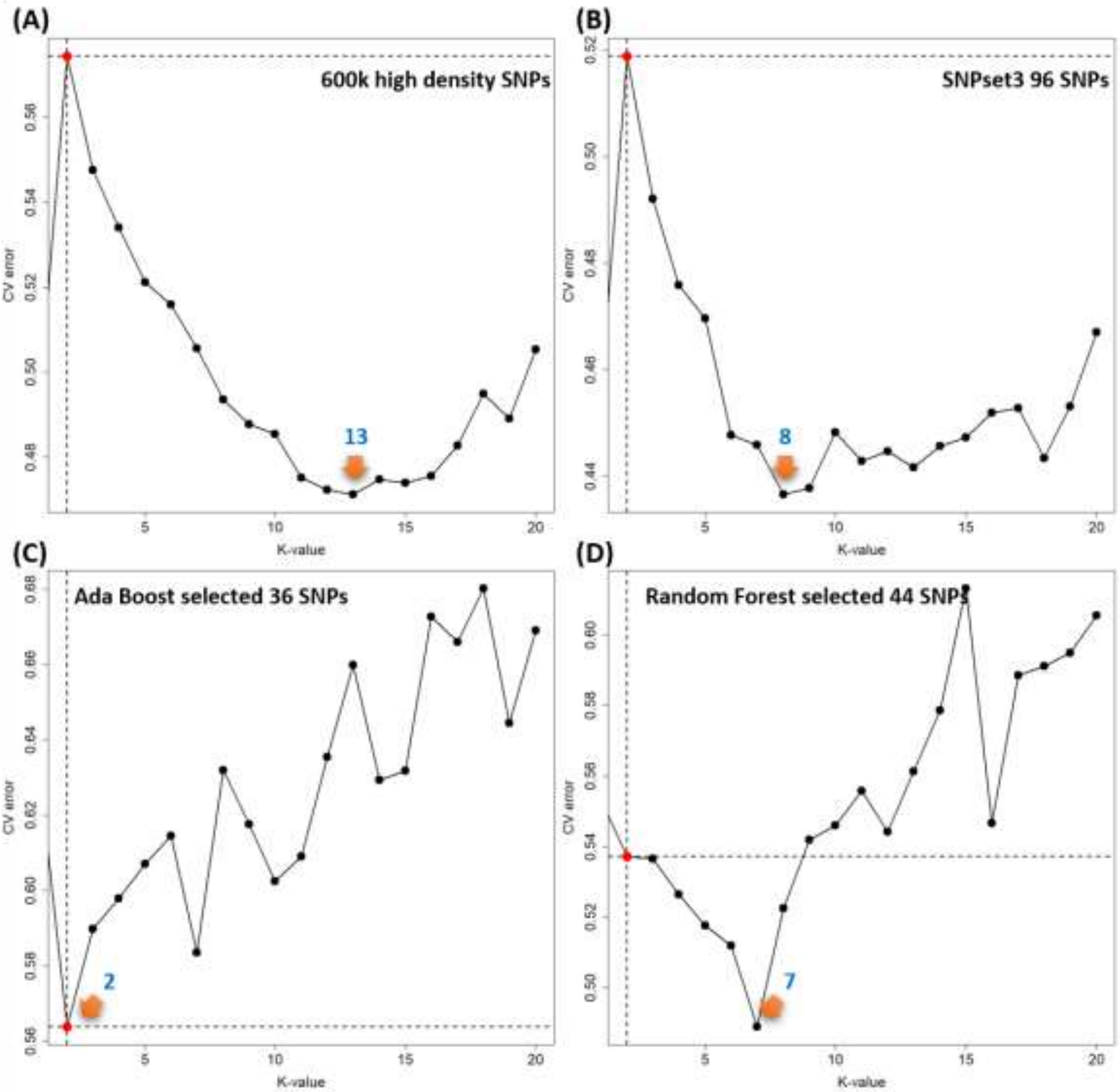

Figure S1. The cross-validation (CV) errors used to confirm the optimal number of genetic components in the chicken population. Two to twenty genetic components were tested, and the optimal number of genetic components that best described the 20 chicken populations was found to be 13 (A). With the selection of suitable SNP markers, the optimal number of genetic components decreased to eight (**B**), two (**C**), and seven (**D**).

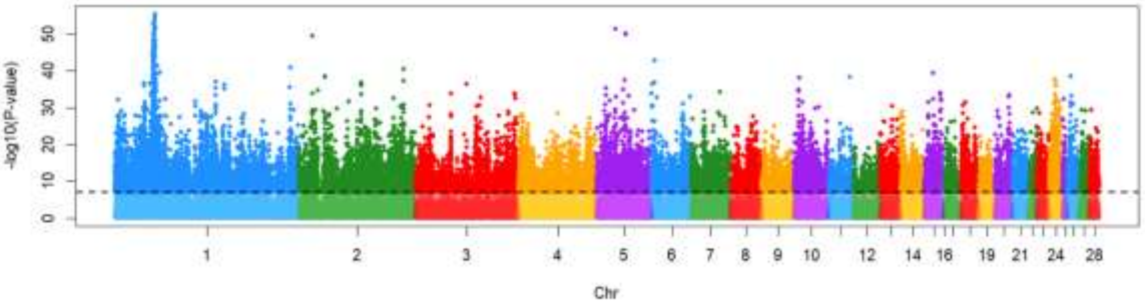

Figure S2. Manhattan plot showing the results of a genome-wide association study (GWAS) for distinguishing case populations (HH, HF, and HY) from control populations (other chicken populations).

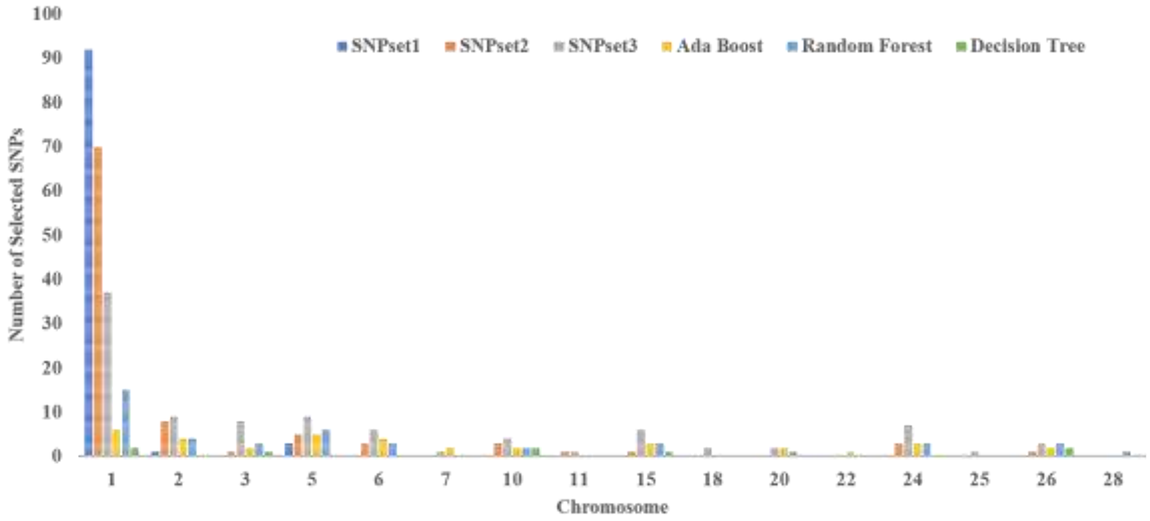

Figure S3. Comparison of the chromosomal distribution of selected SNP marker combinations. SNPset1, which included GWAS-associated SNPs but excluded LD information, harbored 95% of the SNPs located in GGA1. However, SNPs that had undergone 1-LD and 50-LD pruning were distributed in other chromosomes. All machine learning models selected the minimum number of SNP markers from the SNPset3 marker combination, using the feature-selection function.
